# Conducting a prospective evaluation of the development of a complex psycho-oncological care programme (isPO) in Germany

**DOI:** 10.1186/s12913-022-07951-1

**Published:** 2022-04-22

**Authors:** Sandra Salm, Natalia Cecon, Imke Jenniches, Holger Pfaff, Nadine Scholten, Antje Dresen, Theresia Krieger

**Affiliations:** Institute of Medical Sociology, Health Services Research, and Rehabilitation Science, University of Cologne, Faculty of Medicine and University Hospital Cologne, Faculty of Human Sciences, Eupener Str. 129, 50933 Cologne, Germany

**Keywords:** Prospective evaluation, Complex intervention, Care programme development, Mixed-methods, Psycho-oncology

## Abstract

**Background:**

Evaluating the development phase of a complex intervention programme can be challenging. A prospective evaluation approach is presented based on the example of the new complex psycho-oncological care programme isPO (integrated, cross-sectoral Psycho-Oncology). Prior to programme implementation, we examined (1) if isPO was developed as intended, and (2) if it was relevant and transferable into the newly developed psycho-oncological care networks in North-Rhine Westphalia, Germany. Further, we investigated which implementation facilitators and barriers were anticipated and which implementation strategies were planned by the programme designers (multidisciplinary professionals and cancer supporting organizations who developed the isPO programme components and the networks).

**Methods:**

A mixed-methods approach was applied. Qualitative data were collected by quarterly progress reports, interviews and a focus group with the programme designers. Evaluation criteria for document analyses of the quarterly progress reports were developed and applied. Content analysis was applied for analysing interviews and focus group. Quantitative data were gained from evaluating the programme training for the isPO service providers by short written questionnaires that were analysed descriptively.

**Results:**

An implementable prototype of the isPO programme has been developed within 15 months, however no piloting was conducted. The programme’s complexity proved to be challenging with regard to coordination and communication of the numerous programme designers. This was intensified by existing interdependencies between the designers. Further, there was little communication and participation between the programme designers and the prospective users (patients and service providers). Due to these challenges, only context-unspecific implementation strategies were planned.

**Conclusion:**

The required resources for developing a new complex care programme and the need of a mature implementation strategy should be sufficiently addressed. Programmes may benefit from prospective evaluation by gaining insightful knowledge concerning the programme’s maturity and anticipating implementation facilitators and barriers. A mixed-methods evaluation design was crucial for achieving profound insight into the development process.

**Trial registration:**

The study has been registered in the German Clinical Trials Register (No. DRKS00015326) on 30.10.2018.

**Supplementary Information:**

The online version contains supplementary material available at 10.1186/s12913-022-07951-1.

## Background

### Introduction

Cancer is a global health challenge. For the year 2020, 19.2 million new cases and 9.9 million cancer deaths have been reported [1]. A high level of psychological distress was found in over 50% of cancer patients across different tumour entities and at different stages in their trajectory [2]. Over one third of cancer patients show psychological distress considering self-reports within their visit of an oncology centre [3]. Further, psychological and social stress has a negative impact on the general wellbeing and recovery of cancer patients [4–6].

In addition, service providers such as psycho-oncologists, physicians and nurses emphasise the relevance of psycho-oncological care as an integral part of cancer care [7]. Cancer patients who experience less mental health problems are considered to be more compliant to medical cancer therapy [8], indicating that psycho-oncological care can indirectly positively impact the success of biomedical cancer therapy [9]. The management of psychosocial effects of cancer is regarded as a crucial part of comprehensive cancer care [10]. The German National Cancer Plan calls for “need-driven psycho-oncological support for all cancer patients” [11]. In 2014, a German guideline for psycho-oncological diagnosis, counselling, and treatment was published that provides recommendations and instructions for the care of adult cancer patients [12]. However, a nationwide comprehensive psycho-oncological care provision is still missing.

The psycho-oncological care provision gap in Germany should be diminished by developing, implementing, and evaluating a structured and need-driven psycho-oncological care programme.

We aim to outline the benefits of a prospective evaluation and to provide a useful methodological approach. We demonstrate this with the prospective evaluation of the development of the complex care programme isPO (integrated, cross-sectoral psycho-oncology), in order to stimulate the practical application.

### The integrated, cross-sectoral psycho-oncology (isPO) project

The German *integrated, cross-sectoral psycho-oncology (isPO) project* addresses the beforementioned international and national requirements, guidelines and goals [6, 11, 12] towards closing the care provision gap and integrating psycho-oncology into cancer routine care [13]. The aim of isPO is twofold: (1) reducing depression and anxiety in newly diagnosed cancer patients within 12 months after diagnosis, and (2) offering a psycho-oncological care programme for comprehensive implementation into nationwide cancer care. It consists of two parts: care programme and study. The Innovation Fund (IF) of the German Federal Joint Committee is financing isPO between 10/2017–03/2022.

Within the *isPO care programme*, newly diagnosed cancer patients can seek psycho-oncological care from the time of diagnosis until up to 12 months afterwards. On the basis of the patients’ distress and individual needs, as assessed by different instruments (e.g. Hospital Anxiety and Depression Scale), personalized support is offered according to the stepped-care approach [14]. Psycho-oncological care is delivered parallel to the oncological treatment. The patients are supported by a multidisciplinary team, consisting of isPO case managers, isPO onco-guides (cancer survivors working as volunteers providing basic psychosocial information), psychosocial professionals (social workers responsible for psychosocial care), and psychotherapists. The specific care services and roles are described in detail elsewhere [15].

**Fig. 1:**
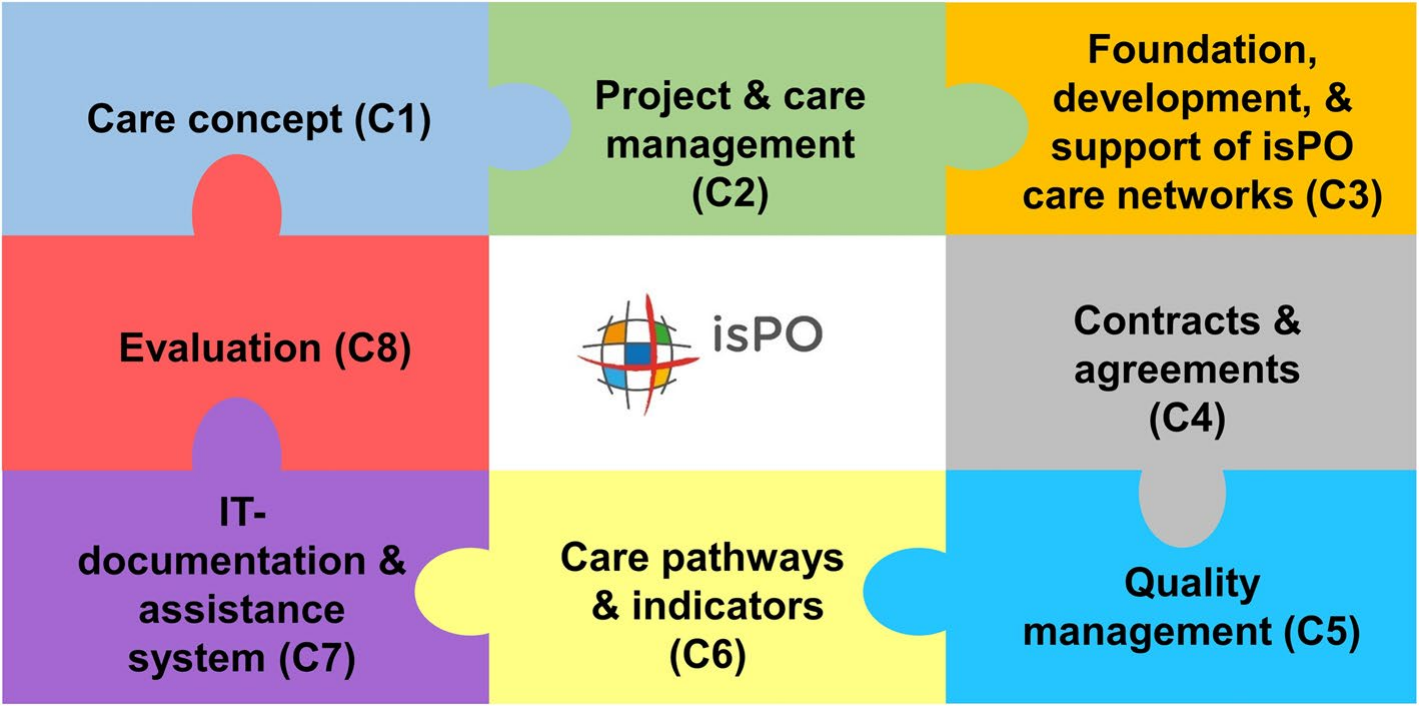
The eight components of the isPO care programme

The isPO programme is a complex intervention [16]. It comprises of eight components (Fig. 1) that needed to be developed, implemented and tested by different consortium partners during the project (see Additional file 1).

The programme was implemented in 2019 in four especially established cross-sectoral care networks in North Rhine-Westphalia, Germany. They respectively consist of at least one certified cancer centre hospital that cooperates with local oncological out-patient practices.

isPO is intended to be implemented into routine cancer care, if positively evaluated in the summative evaluation. Therefore, the isPO programme is accompanied by a *study,* enabling an internal and external evaluation of the programme as required by the funding organisation IF [17]. The external evaluation is conducted by the independent Institute of Medical Sociology, Health Services Research, and Rehabilitation Science, University of Cologne (IMVR). The IMVR team accompanies the entire project, but is not actively involved in the development, implementation or care provision of the programme. In order to optimise the programme, evaluation results are continuously fed back to the project management team, who then decides if action needs to be initiated, and in some cases directly to the respective programme designers.

**Fig. 2:**
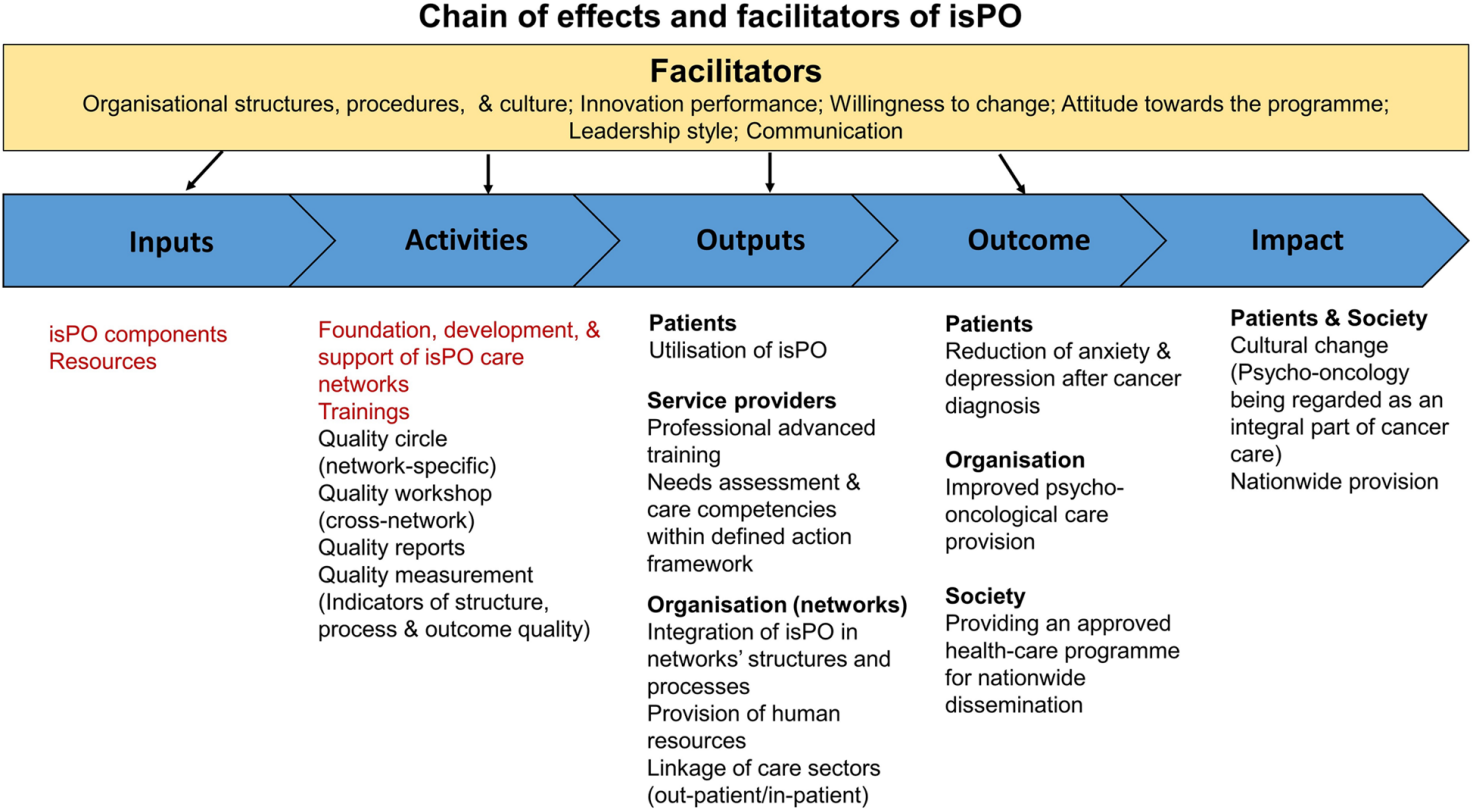
Chain of effects and facilitators of the isPO care programme. Adapted from [18] Anderson et al. (2011); [19] Damschroder et al. (2009). Aspects that were examined within the prospective evaluation are highlighted in red

The content-related basis of the evaluation concept follows a logic model (Fig. 2).

**Fig. 3:**
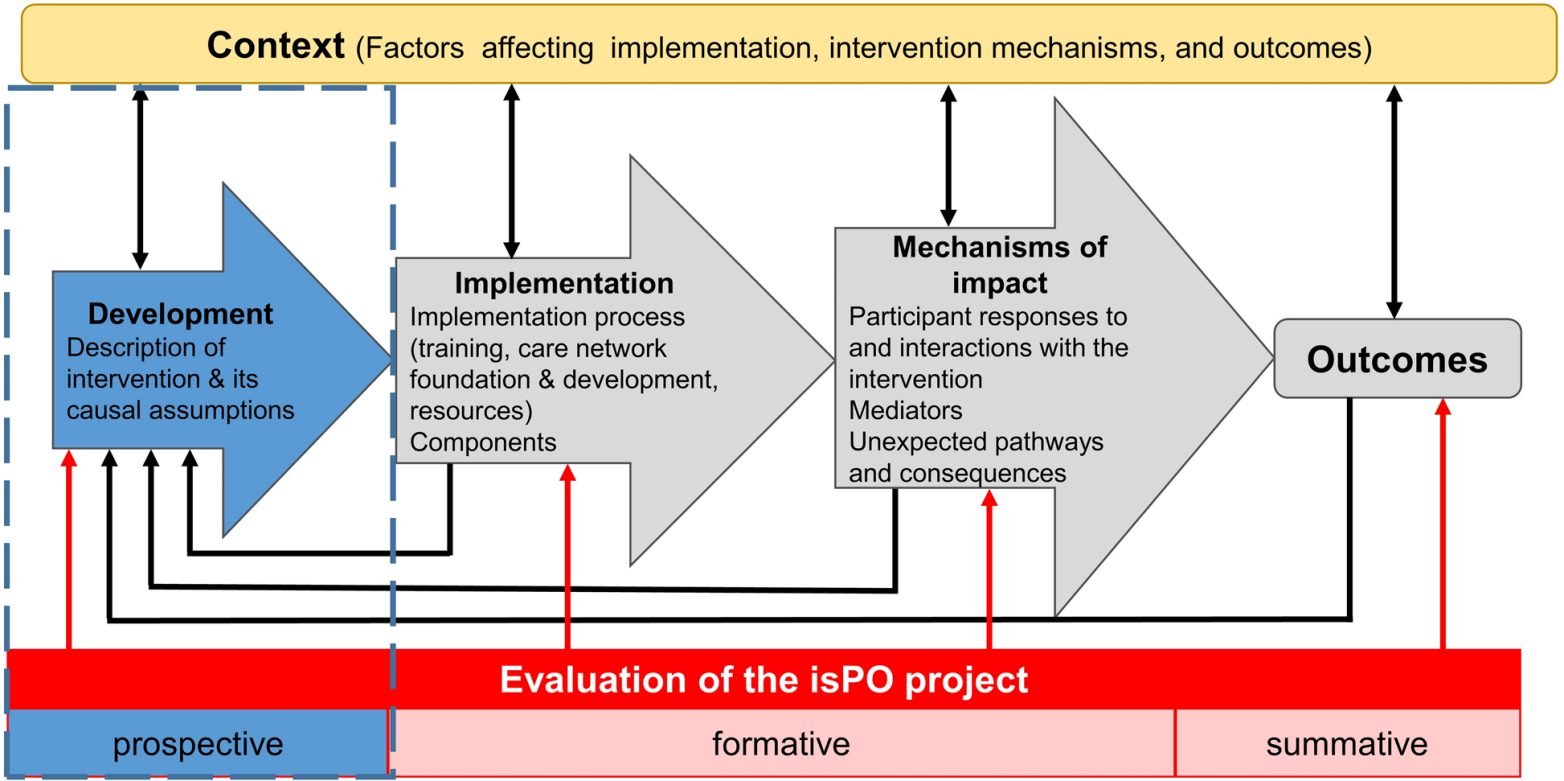
Tripartite process-oriented evaluation design of the isPO programme. Adapted from [20] Moore et al. (2015)

The evaluation team conducts a tripartite process evaluation (Fig. 3), based on the Medical Research Council (MRC) Framework for analysis and evaluation of complex interventions [20]. The constructs of the Consolidated Framework for Implementation Research (CFIR) [19] defines several facilitators (Fig. 2, overarching part) for a successful implementation.

For the isPO-programme, the specific chain of effects is examined, especially its inputs (components), activities concerning programme implementation (e.g. care network foundation), and outputs at patient, service provider, and organisational level.

This article focuses on the prospective evaluation and its methodological approach. The entire study design for the evaluation of the isPO programme is described in detail elsewhere [13].

### Objectives of the prospective evaluation

This article demonstrates the prospective evaluation of the isPO programme, also referred as developmental formative evaluation [21]. The aim of the prospective evaluation was to assess the relevance and transferability of the isPO programme prior to its implementation. With regard to the inputs and activities in Fig. 2, the prospective evaluation is guided by the following research questions (RQ):

RQ1: Were all isPO programme components developed as intended according to the project proposal?

RQ2: How were the isPO care networks recruited and developed?

RQ3: How did the isPO programme designers experience their communication and cooperation within the project?

RQ4: Which implementation facilitators and barriers did the designers anticipate and what was their implementation strategy?

RQ5: Does the concept of the isPO programme appear to be consistent and useable?

## Methods

This prospective evaluation was conducted by a multi-disciplinary evaluation team with expertise in Health Services Research, Public Health, Psychology, and Sociology.

To assess the relevance and transferability of the isPO programme before its implementation, all developed isPO components were examined.

**Fig. 4:**
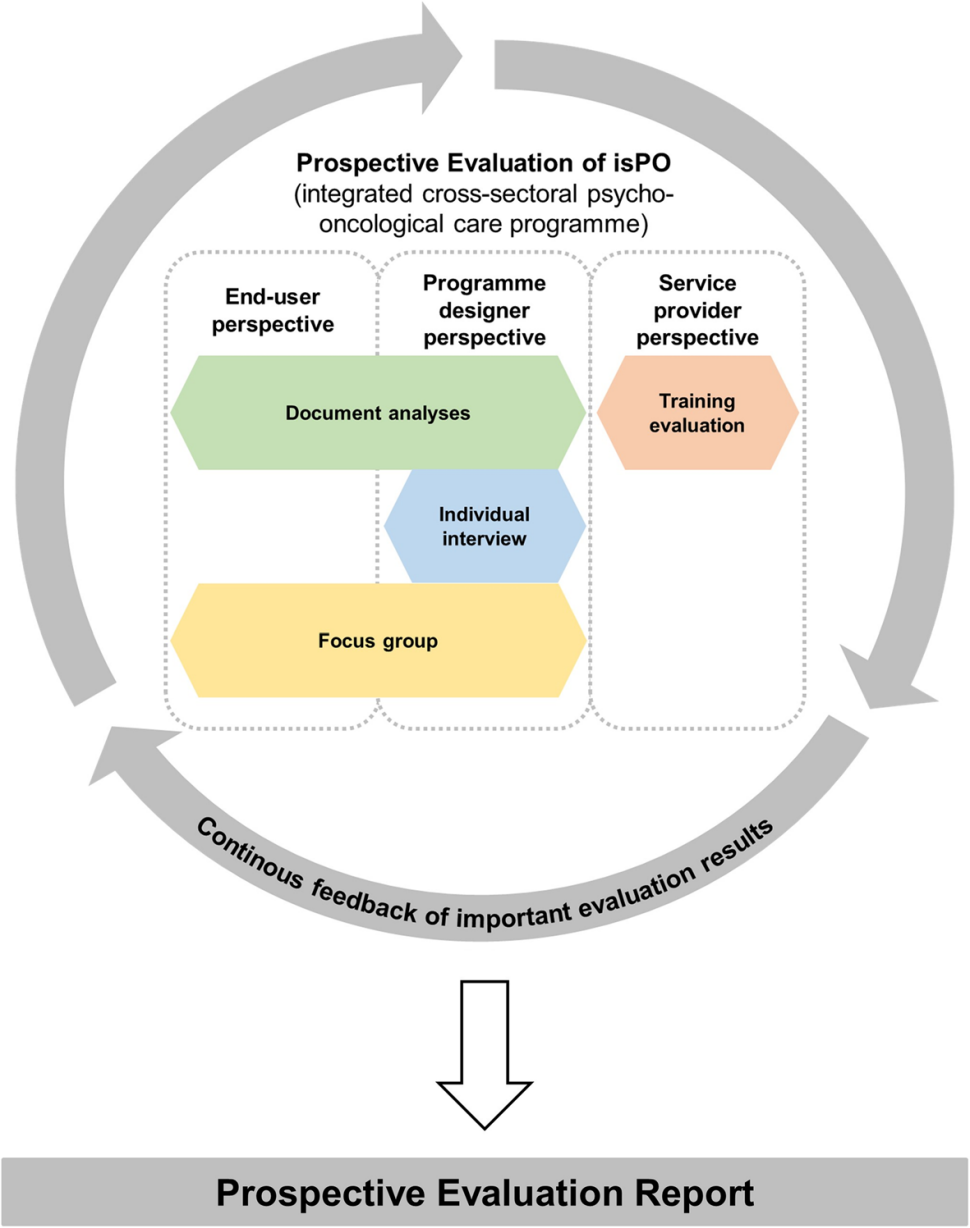
The mixed-methods design of the isPO programme’s prospective evaluation

A QUAL-quant mixed-methods design [22, 23] was chosen (Fig. 4) in order to gain rich insight into the stakeholders’ experiences as well as the development and working process itself. In order to assess the programme’s development and its readiness for implementation, knowledge was gathered from three perspectives: (1) end-user (cancer patient), (2) service provider and (3) programme designer (Fig. 4). The House of Cancer Patient Support Associations of Germany (HKSH-BV) represented an overarching patient perspective on the programme’s development, as it is the German umbrella organisation for ten cancer self-help organisations with approx. 1,500 self-help groups and is familiar with consulting and supporting research projects in cancer care. Persons within the HKSH-BV and its ten affiliated organisations are typically cancer patients, survivors or caregivers who provide peer support or engage politically as patient representatives. Employees of the HKSH-BV with long-term experience in representing cancer patients consulted the isPO project. The representation was necessary as patients could not have any experiences with the programme at that phase of the project. This also applies to the service providers, which is why they were asked about their experience with the isPO-trainings that were conducted at the end of the development phase, just before implementation started. The majority of data on the programme’s development process therefore originates from the programme designer’s perspective.

Four different data collection methods were applied: (1) document analyses, (2) individual interviews, (3) focus group interview, and (4) training evaluations (Fig. 4).

**Fig. 5:**
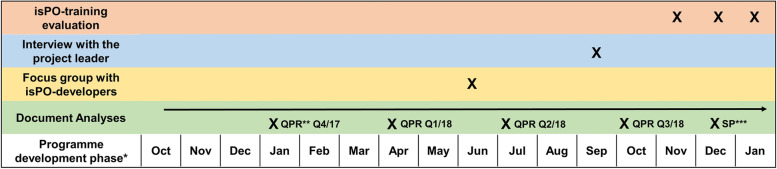
Timeline of the used data collection methods in the prospective evaluation of isPO. Legend: *Programme development phase started in October 2017. Implementation and patient recruitment started in January 2019 in one isPO care network. The following months the other three care networks also started recruitment. **QPR Quaterly Progress Reports that are written by all programme developers. ***Statement paper of the House of Cancer Patient Support Associations of Germany

The focus on qualitative data collection allowed a more elaborate insight into the programme’s development process and therefore it was helpful to gain comprehensive knowledge. However, quantitative data collection was used to evaluate the service providers experience in the pre-implementation isPO-training. The resulting mixed-methods design allows a multi-perspective and detailed data collection to answer the posed research questions [24]. Figure 5 shows the applied data collection methods in the programme development timeline.

### Document analyses

The document analyses’ aim was to gain deeper insight into the programme designers’ work and perspective within the project. We evaluated if: a) the isPO programme components (Fig. 1) were developed as intended (RQ1), b) the programme is consistent and usable (RQ5), and c) how the isPO care-networks were recruited and developed (RQ2). For this purpose, it was necessary to analyse three different document types: 1) contracts (e.g. regulating the provision of the isPO care services in the care networks), 2) project-associated documents that help to understand the programme’s development (e.g. SOPs for patient recruitment), and 3) quarterly progress reports of all programme designers.

To enable the intended realization of psycho-oncological care in the isPO care networks, it was necessary to establish a contractual framework. These contracts were evaluated by the evaluation team by: 1) comparing them with the project proposal, in which the legal parameters for the contractual framework of isPO were already strategically planned and set out, and 2) by assessing if the contracts allow a comprehensive dissemination of the isPO programme into the networks, but also potentially into nationwide routine care.

The evaluation team also assessed certain project-related documents that are relevant for a successful implementation or evaluation of the isPO programme, e.g. training documents or protocols of project meetings.

The consortium partners responsible for the development of the respective programme components were requested to report about their work progress on a regular basis, in so-called quarterly progress reports (QPR). A semi-structured QPR frame was set up by the evaluation team, allowing for consortium partners’ adaptions to individual requirements.

Fifteen QPRs from six consortium partners were evaluated with a specially developed criteria catalogue (Table 1). The QPRs and their systematic evaluation helped to gather information on the isPO programme’s development, progress and scientific foundation, as well as information concerning the designers’ expectations and identification with their role in the development and implementation of the care programme.

**Table 1:** The isPO QPRs Evaluation Criteria Catalogue

Evaluation structure	Evaluation criteria	Explanations and in-depth evaluation criteria
**General Information**	Author	
	Structure	
	Orientation on deadlines	
	Orientation on the template	
	Annexes	
**Theme-specific evaluation**	Role in the project	Description of the task area Comparison with the project proposal Role in the project becomes apparent (consortium partner knows own role and can differentiate it from other roles) Application orientation / "view for practice" (definition and description of target groups; if known, it is described)
		Classification of the subtasks within the area of tasks Is the subtask visible as part of the task area (embedded vs. subtasks)
	Scientific / specialist background	Presentation and justification of the basic principles (if applicable: guidelines, standards, laws, theories, experience, etc.) Context of the tasks comprehensible Comparison with project proposal
	Goals	Project reference, embedding in the task area (if necessary, use table) Milestone vs. additional goals
		Definition of the goals: specific, measurable, achievable, realistic, terminated (‘*SMART*’ principle)
	Ways to achieve aims / measures	Explanation of content and transparent justification, comprehensible achievement of goals (can this measure achieve this goal?)
		Representations are intersubjectively comprehensible
		Type of measure (sub-measure)
	Results	Transparent presentation of the results (partial results)
		Were the (quarterly) goals achieved? How many goals are there with no result? Are there goals without a result?
		Existence of deviations Description of deviations Evaluation and handling of deviations for the achievement of individual goals and milestones Measures and solutions for the deviations
		Planned changes Description of the changes Evaluation and handling for the achievement of goals and milestones Measures and approaches regarding the changes
	Further procedure / future orientation	Description of the planned milestones and goals (and planned measures, if any)
		Comparison with project proposal Comprehensible justification for additional goals
	Focusing and prioritising of topics (qualitative)	Which topics are in focus (occur how often in the sense of unconscious prioritization)
	Cooperation with the consortium partners (dependencies etc.)	Scheduling / project meetings
	Implicit, conscious or unconscious communication content	Institutional traces (author / non-writer) / institutional exhibition (Goffman, 1972), Personal and institutional intentions in the presentation—documentary method
		If applicable, which topics are not mentioned (or not addressed actively)
	Contradictions	Text vs. traffic light (milestones vs. task description)
	Orientation towards guideline *S3 Psycho-oncology* (overarching embedding)	
**Document comparison ("conversation between documents"; intra and inter)**	Contradictions	
	Cooperation (mutual naming of the consortium partners)	
	Timeline: Course of a consortium partner (internal)	Comparison of the subtasks Do the tasks build on each other? Are the tasks embedded?
**Conclusion**	Concise assessment as a consequence of previous analyses	

The evaluation team compared the respective work results with the initial project proposal and prevailing healthcare guidelines and laws. Specific requirements were defined for each programme component by the project proposal. These should be met by the programme designers by the end of the development phase.

Additionally, a statement of the HKSH-BV was included in the prospective evaluation as an overarching patient perspective on the programme’s development.

### Interview with project leader

In order to gain the project management’s perspective, an interview with the project leader was conducted by an evaluation team member at month 12 of the development phase (Fig. 5). It aimed to supplement the respective QPRs, but also to gain more profound explanations on the isPO programme’s conceptual framework.

Additionally, fundamental topics, such as the conceptual framework of the programme and its implementability, were addressed. The interview lasted two hours, was audio recorded, transcribed and thematically analysed [25]. The results were included in the evaluation of the QPRs.

### Focus group and telephone interview

To gather the programme designers’ and prospective end-users’ perspectives, one focus group and telephone interview were conducted. The aim was to identify: a) possible implementation facilitators and barriers (RQ4), b) if the programme designers anticipated these factors (RQ4), c) if the programme designers established implementation strategies (RQ4) and d) how the programme designers experienced communication and cooperation within the isPO project (RQ3). Purposeful sampling was applied by inviting at least one person of each programme designer group involved in the programme design (sub-project leaders) [26]. In all, seven representatives participated in the focus group. The cancer patient perspective was represented by the participation of the HKSH-BV. Six programme designers, who were respectively responsible for developing a specific programme component, attended the focus group (one to two representatives per programme component, Fig. 1). The representative of the IT-documentation and assistance system CAPSYS^2020^ was not able to attend, but was willing to participate in a telephone interview, post focus group.

The focus group interview was conducted nine months into the programme´s development phase (Fig. 5). The individual telephone interview followed shortly after.

The focus group was conducted in the premises of the IMVR by two evaluation team members (one main moderator and one co-moderator). Both, the focus group and the telephone interview were conducted in a semi-structured form by using interview guidelines. After conducting the focus group, the interview guideline was augmented with topics that arose during the group interview, which also allowed the interviewee of the phone interview to comment on it (Table 2). The focus group lasted 115 min and the telephone interview 16 min.

**Table 2:** Topics included in the interview guideline for the focus group and telephone interview

Topics in the focus group guideline
Cooperation between programme designers
Cooperation with and perception of the care networks
Implementability of the programme
Implementation strategies
Facilitating and hindering factors for programme implementation
Activities to achieve project goals
Programme’s potential to be disseminated into national care structures
Topics additionally included in the telephone interview
Information flow within the project
Perception of service providers’ acceptance towards the care programme during CAPSYS^2020^ training sessions

The focus group and the interview were audio recorded and transcribed. Content analysis was performed [27, 28], assisted by the MAXQDA software (version 12.0). The coding and analysing process was conducted by the same evaluation team members that collected the data. First, coding was conducted independently. Next, codes were discussed, the transcript recoded, discussed again until a consensus was reached and the final coding system was decided upon (see Additional file 2). Coding was at first based on the guideline leading questions (deductive), however, during viewing the transcript, codes were also derived inductively [27].

### Quantitative evaluation of the isPO-training

To gain the perspective of the service providers before the programme’s implementation, the isPO-training was evaluated using short written questionnaires (Fig. 4). The service providers’ training courses were conducted mostly as frontal lectures at the end of the development phase by the respective programme developers (Fig. 5). First, all service providers received an overall introduction into the project that lasted approx. three hours, followed by three hours training regarding their specific role in the isPO service provision. Additionally, special training for the newly developed IT documentation and assistance system CAPSYS^2020^ were realized in face-to-face training and with the help of videos, which were uploaded on an e-learning platform. Lastly, as basis for their certification, special 5-h-training was conducted for the isPO onco-guides, who are cancer survivors, and not professional service providers. Their training was conducted as lectures as well as role-playing exercises for conversation conduction.

The service providers attending the basic isPO-training (7 training sessions with 6 to 13 participants each), filled out an anonymous evaluation questionnaire with 13 to 15 items that was developed by the evaluation team. It was used for each training session and included questions about the comprehensible communication of the following summarized content: project structure, care concept, development and function of the care networks, respective care pathways within the care programme, quality management, patient recruitment, isPO onco-guide concept, and tasks within the personal role in the isPO programme. Furthermore, participants were asked to evaluate, if 1) all questions were clarified during the training, 2) the time frame was appropriate, 3) the trainee was competent and motivated, 4) the training was well organised and 5) they were satisfied with the training. Lastly, participants were asked to suggest improvements.

The evaluation questionnaire for the programme’s IT system training also included system specific questions (see results). For all items, participants were able to rate their (dis-) agreement on a four-point Likert scale from 1 ‘not at all’ to 4 ‘totally’, with an additional ‘don’t know’-option. Descriptive analysis was conducted with SPSS 25 and respectively summarised. The results where then send to the respective training instructors to provide direct feedback.

### Bundling of results in the evaluation report

By the end of the development phase, a prospective evaluation report was written up, including all results in detail (Fig. 4). It aimed to illustrate and interlink the evaluation results from the three relevant perspectives (end-users, programme designers, and service providers) and to draw conclusions and lessons learned from the programme’s development for the following implementation phase.

The report is similarly structured to a standard scientific manuscript, and differentiates between conclusion, lessons learned and recommendations. By this differentiation, we aimed especially for a content condensation and interpretation of the results that was relevant for further programme development and implementation, as well as the evaluation processes themselves. The report was forwarded to project leadership.

## Results

The prospective evaluation results are presented at an end-user, programme designer and service provider level (Fig. 4).

### Document analyses and interview with the project leader (end-users’ and programme designers’ perspectives)

#### Patient perspective (end-user) on the isPO project, represented by the consortium partner HKSH-BV

The HKSH-BV emphasised the importance of psycho-oncology for the care of cancer patients resulting from cooperation between different professions. Three conditions in the isPO programme were very welcome: (1) former cancer patients are trained and included as volunteer isPO onco-guides and complement the professional isPO support team with the peer support, (2) the cancer self-help is represented contractually for the first time in Germany, and (3) the management of the isPO onco-guides’ care provision is financially covered. In addition, there was a quality assurance for the care provision by isPO onco-guides through defined requirements for the certification as an isPO onco-guide. This includes a special training, a conflict of interest statement, and a commitment statement.

The overcoming of sector boundaries (in- and out-patient) is perceived as a fundamental, patient-relevant feature of the isPO programme. This ensures continuous psycho-oncological care, even in the case of a transferal from one sector to the other within medical cancer care (e.g. from in-patient to out-patient care). The clear definition of care pathways, with the deposit of necessary documents, is seen as an important measure for a high quality of care, which in turn is decisive for patient safety. Due to the development of a comprehensive care programme, the isPO programme is considered by the end-users (HKSH-BV) as sustainable.

The HKSH-BV reports that, in addition to the advisory function, other tasks were taken on during the project year. The consortium partner engaged in developing the isPO onco-guide concept, recruiting former cancer patients and training them as isPO onco-guides.

The cooperation with the other consortium partners is perceived as "close, fruitful and appreciative". The high level of commitment of all project members is valued.

#### Programme designer perspective on the isPO project

In order to summarise the programme's development process for each programme component, the actual working achievements are illustrated in comparison with the aims according to the project plan in additional file 3. Each objective is assigned to the corresponding working result and further activities beyond the project plan are displayed. Due to the programme’s complexity and delays in the development process, creating the isPO prototype took 15 months (10/2017–01/2019).

#### Care concept (C1)

**Fig. 6:**
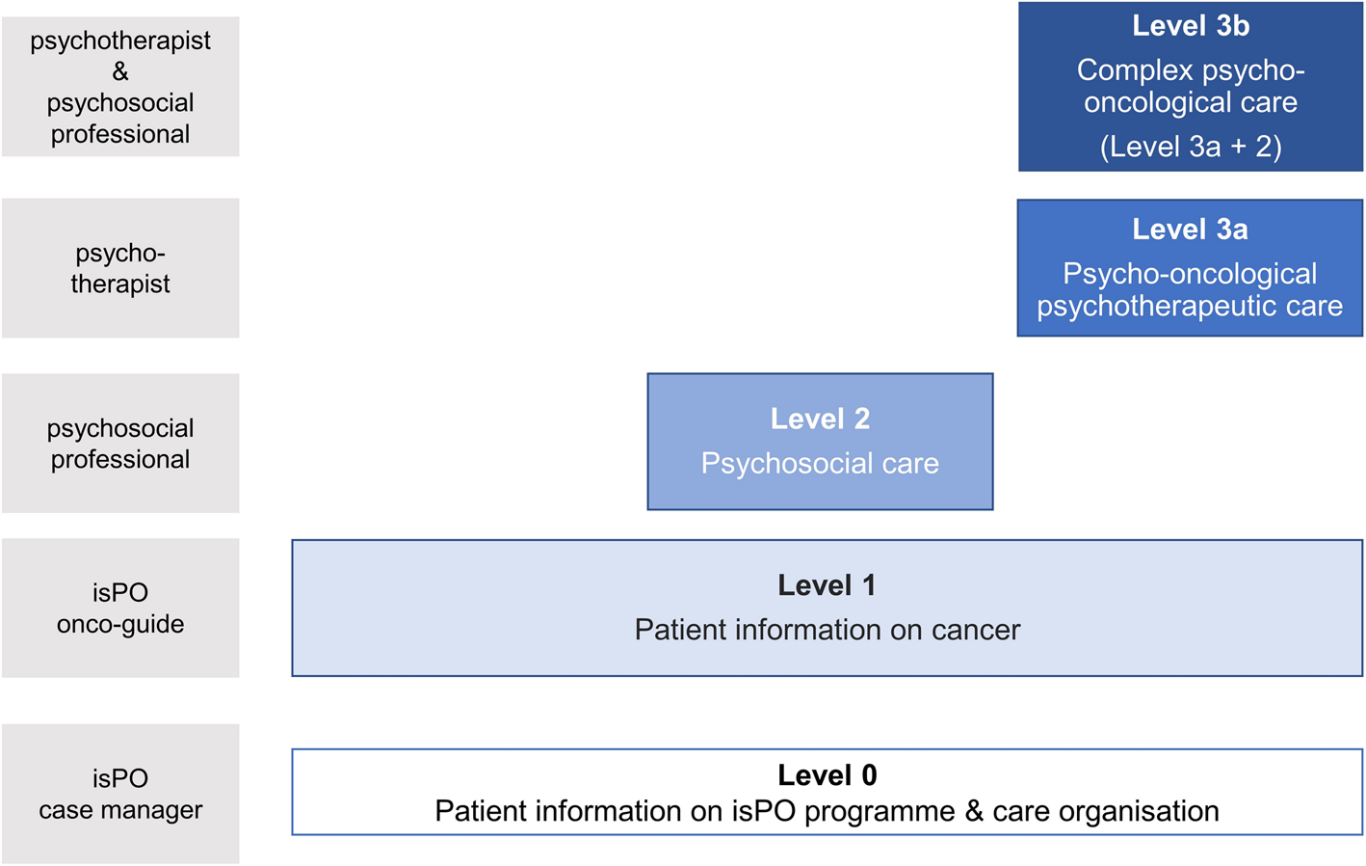
Care levels of the isPO programme and the service providers working at each level

The scientific basis of the care concept was developed. Consequently, the isPO care provision can be offered according to patient’s needs at different care levels (Fig. 6; see detailed description of the care concept elsewhere [15]), to which different measures and service providers are assigned.

Due to the short timeframe for the programme development, interdependencies among the consortium partners, and partially insufficient communication, the complete care concept has not been written down comprehensively at the end of the development phase. This was postponed to the start of the implementation phase.

#### Project & care management (C2)

A document control system was created and elucidated, as along with the necessary organisational structure for managing care in the care networks. Regular meetings with consortium partners and the steering committee were established.

#### Foundation and development of isPO care networks (C3)

In addition to the University Hospital Cologne, three more networks were recruited to cover a broad spectrum of different population and care structures. However, these various prerequisites lead to different states of network establishment at the end of the development phase. Based on the hospitals’ scope of care and personnel resources, it was assumed that the planned recruitment goals could be achieved whilst providing other patients with the hospitals’ regular psycho-oncological care in parallel, albeit, with significantly increased effort. Altogether, the four care networks were developed within 16 months.

#### Contracts & agreements (C4)

All necessary contracts and agreements for care provision have been signed. The “isPO care contract” has achieved an innovation in the German healthcare system, especially with regard to the integration and financing of psychosocial care and organisation of self-help services (isPO onco-guide).

#### Quality management (C5)

A beta version of the project-related quality management manual was produced. Quarterly internal care network quality circles and cross-network quality workshops were planned, aiming to involve the care networks in the optimisation process (participatory quality development approach).

#### Care pathways & indicators (C6)

Basic SOPs for care levels 0 to 3a (Fig. 6) and care pathways for care level 0 to 2 were modulated. The SOPs will be further elaborated, adjusted, if necessary, and finalised as part of a continuous improvement process during implementation in practice.

#### IT-documentation and assistance system (C7)

**Fig. 7:**
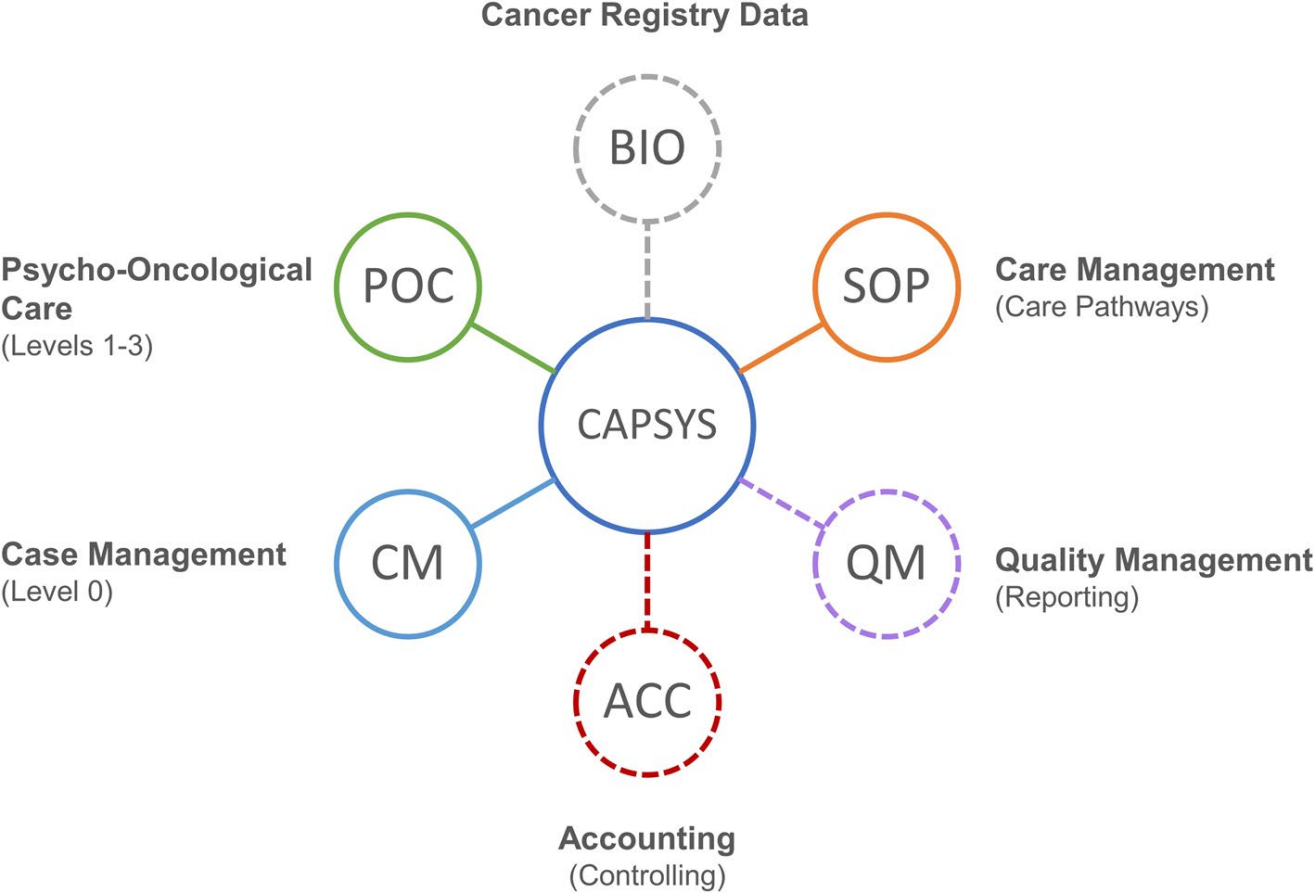
Documentation and care management elements of the ‘Computer-based Assistance System Psycho-Oncology’ (CAPSYS^2020^). Functions that have not been finalised at the end of the development phase are presented in dashed lines

Due to interdependencies between consortium partners, some important goals were not achievable. The development of the three areas "accounting", "quality management" and "cancer registry data" (the latter being used for evaluation purposes) remained immature (Fig. 7). Therefore, a paper-based documentation will be utilised during the initial transitional period in the implementation phase, and later transferred into the IT system.

#### Evaluation (C8)

In order to enable a comprehensive study as well as external evaluation of the care programme an isPO data warehouse was set-up, and a comprehensive data protection concept was developed. Due to delay in the development of quality indicators (C6), the programming of the care statistics was not carried out in the development phase. To partially compensate this milestone deviation, extracts from the cooperation agreements were used to derive test quality indicators and thus build up the processes of statistical calculation and data preparation.

### Focus group and telephone interview (end-users’ and programme designers’ perspectives)

**Fig. 8:**
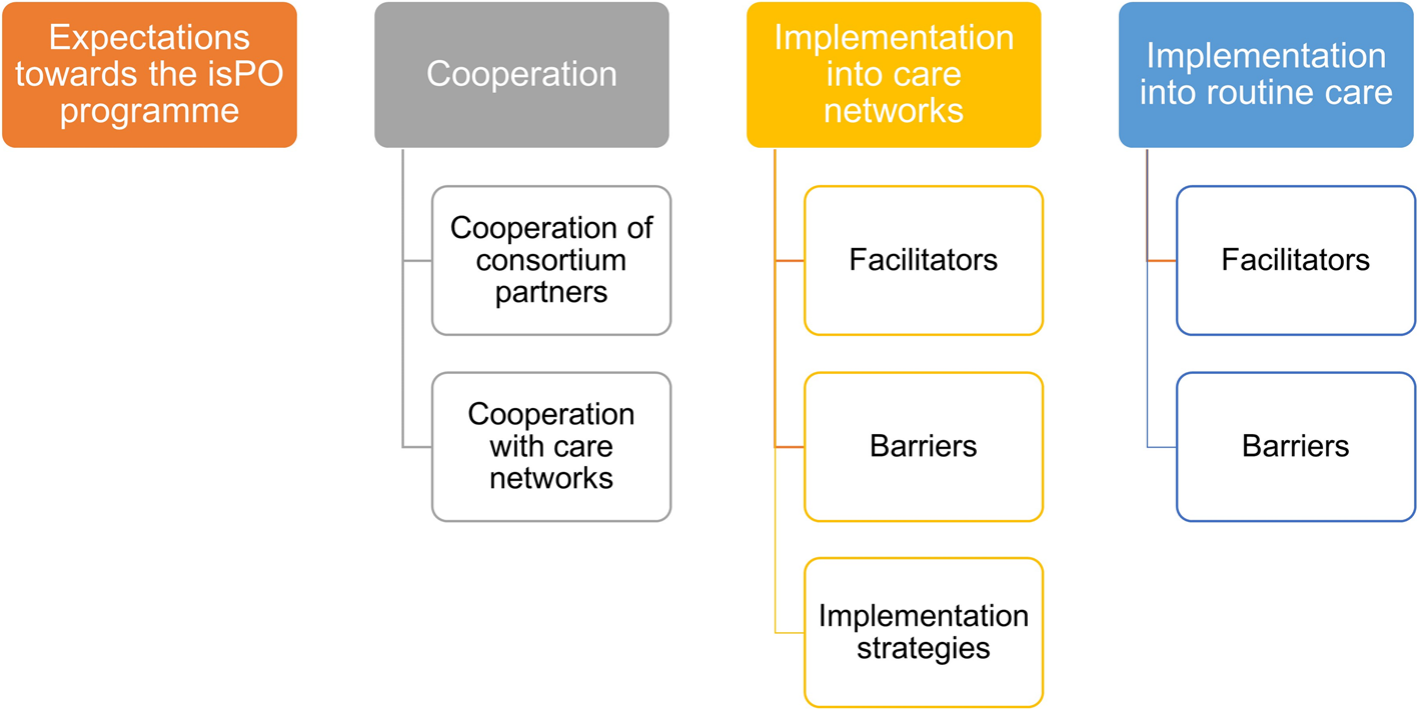
Overview of the developed core categories for coding the focus group and the telephone interview

The results are presented in accordance with the four core categories: expectations, cooperation, implementation into care networks, and implementation into routine care (Fig. 8).

#### Expectations towards the isPO programme

Both, the patients’ representatives and the programme designers expect that isPO implementation will lead to evidence-based, structured, improved and effective psycho-oncological care. In addition, they expect that it will contribute to: (1) optimising the current psycho-oncological care structures, and (2) including psycho-oncology in the billing system and in the catalogue of services of the statutory health insurance. This goes hand in hand with the expectation that psycho-oncology will be strengthened in its position as an integral part of cancer therapy.

#### Cooperation of consortium partners

All participants described the cooperation amongst themselves as constructive, and communication at a personal level as good. The working groups involved in the isPO programme’s conceptual design, in particular, were in close contact with each other.

All partners mutually appreciated the very high level of commitment of each partner. They described this fact as motivating for their own work. It reflects that everyone was aware of the importance and scope of this project. Despite the high level of commitment, concerns were expressed as to whether the project tasks could be completed within the timeframe. The workload was perceived as emerging and very high.

*"... we are now facing the challenge, especially in the first year, of bringing up a complex programme in a very short time on many different levels and dimensions. And this with many instances or with many different partners."*

The timeframe was perceived as an obstacle, since several interdependencies between the consortium partners exist. For their own progress, they were reliant on information from and the results of the work of others.

The internal communication within the project was viewed critically by most partners. A lack of a “*superordinate unit*”, distributing relevant information to all participants, was perceived. This was stressed especially by those partners who were not directly involved in the programme conception. They would like to see "*a denser flow of information*" and reported that they would receive completed project steps "*at best by chance*".

#### Cooperation with care networks

So far, the cooperation with the care networks was almost exclusively with the consortium partner responsible for the care networks’ development. The other partners were not engaged with the care networks during the programme’s development.

The care network developers described the cooperation as intensive. Regular monthly working meetings took place. In addition to providing information about the project and its implementation, there was a need to increase the intrinsic motivation of the care networks to get involved in the isPO programme. It was experienced that reservations and concerns (see subsection ‘barriers to implementation into care networks’) had to be dealt with. Therefore, information was passed on carefully and "*diplomatically*" in order to convey a realistic picture of the requirements, but not to trigger a feeling of being overwhelmed that might lead to resistance. All programme designers found it important to be open to criticism and the experiences of the care networks, as this will support the implementation in practice.

#### Facilitators of implementation into care networks

The programme designers perceived the acceptance and motivation of the care networks’ service providers as crucial for the isPO programme’s implementation. During the care networks’ development process, a pronounced interest in the project and an increased level of motivation were noticeable. Nevertheless, it was found to be important to continuously promote the service provider’s acceptance, as this might facilitate the implementation. This can be done by emphasising both the importance of the project, as well as the role and contribution of each individual in the care networks. Also, the importance of structuring and formalising the psycho-oncological documentation should be continuously communicated, especially to increase the acceptance of the new computer-based documentation and assistance system CAPSYS^2020^.

"*...if you provide regular and modern care today, then first of all you have to document this care properly and secondly, […] including the healthcare system, that we also have to strengthen the process orientation in care ...*"

IsPO is perceived as a patient-oriented programme. Despite the fact that it is assigned to a specific care level, each screening should be used to check whether patients are receiving adequate care.

Considering the different structural situations before the implementation, for example personnel capacities, might be central for the implementation process. Existing care network structures with regard to diagnostics and documentation may facilitate its implementation.

The focus group participants hoped that the monetary incentives, given for care within the isPO programme, would offer a reimbursement for the additional efforts. IsPO enables the refinancing of psycho-oncological care services for the care networks for comprehensive psycho-oncological care.

Due to the participatory quality development approach, participants also perceived a high potential for the implementation phase. It was pointed out that both, the structures and the tasks of the different roles in the care network were clearly defined, which favours implementation.

#### Barriers to implementation into care networks

Low acceptance and motivation of the care networks have also been seen as a barrier to the implementation process, provoking a negative attitude towards isPO. Thus, resistance of service providers was perceived as possible, due to associated change processes in their respective work place. The project specifications could lead to restrictive feelings among the service providers with regard to previously established working processes (vs. new isPO processes) as well as therapeutic freedom in the form of a "*forced corset*".

The participants assumed that care networks might perceive isPO as a “threat” to their internal care structures, should the implementation of isPO replace these. However, such fears could be refuted at the level of therapeutic freedom, since isPO care is only intended to provide a "*framework*", within which the service providers can "*continue to choose the intervention themselves*".

It is important to consider the needs and experiences of the service providers so that they do not feel "*overwhelmed*". Another reason for resistance might be attributed to the study part of the project. Since certain procedures are linked to the fact that isPO is not only a new care form, but is accompanied by a study, service providers may feel restricted in their scope of action.

"*...that you simply say that in this project it has to be constructed in a certain way, which we know does not correspond to real life in all places. So, to remove the fear, that this is how it should be done in the future.*"

Service providers may see the fact that isPO requires new processes as an obstacle to patient-oriented work.

Moreover, it was stated that certain scenarios had not yet been conclusively clarified and that the care networks had, up until now, little detailed programme knowledge. This may lead to uncertainties in the care networks during implementation.

Reservations from the management (e.g. higher personnel costs) could influence the implementation process, acting as a barrier.

#### Implementation strategies

The training for all service providers and the availability of target group-specific manuals as a written form of the programme’s concept were outlined as essential implementation strategies. It was important to create a balance between detailed description of the programme’s content in the manuals and its scope, as not all care scenarios could be covered. Above all, the isPO manual and quality management manual are intended to provide guidance for work in isPO beyond the training. In addition, CAPSYS^2020^ is supposed to guide the service providers through the process. During the discussion about implementation strategies, the participants focused on possible communication strategies and how to deal with care networks’ resistance. They stated that a communication interface between programme designers and care networks was needed:

"*...whatever that is, we need a feedback system.*"

Collecting similar questions and distributing information to all networks might be solved by this platform. The designation of a contact person was considered useful to support and accompany the care networks in the "*first orientation phase*" of the implementation.

#### Implementation into routine care

Since the isPO project addresses a field of care that is currently insufficiently provided in Germany, its potential to be implemented in routine care was estimated as high. Furthermore, its unique design, which according to the stepped-care approach addresses patient needs, enforces this notion. The structured nature of the isPO programme was seen as a facilitating aspect for the implementation into routine care, as it

*"...will generate significantly greater acceptance, also on the part of the medical professions, but also on the part of politics, and thus integration into the health insurance remuneration system, ..."*

It was considered to be of central importance to already become politically involved during the project period in order to promote nationwide adoption after project completion.

Moreover, it was perceived as necessary not only to prove the interventions’ effectiveness (end-user level), but also to identify and consider as many implementation factors as possible (e.g. attitude of the service providers, acceptance of the patients towards the programme). A high-quality evaluation could shorten the time required for assessment by key institutions and thus accelerate adoption, so that the care provision gap after project completion is kept short.

However, a potential conflict was perceived at the professional political level since psychotherapists’ position will be strengthened by isPO, but relevant decision-making committees are more occupied by physicians. At the level of national psycho-oncological care structures, fears were expressed that bureaucratic processes would impede rapid adoption into routine care.

Regarding the programme’s implementation into routine care, uncertainties were expressed, that there were currently no plans of the funding organisation (IF) on how to practically organise a comprehensive implementation of funded new care forms like isPO. This raised the question:

"… will the conditions be created to ensure that a new care form … in Germany in the field of psycho-oncology … will continue to be possible in the future…"

Methodological aspects might also impede the nationwide adoption. If the isPO project had methodological weaknesses, for example due to the fact that the care programme was not carried out in accordance with the concept or lack of data and inadequate analyses, there is a risk of a negative evaluation outcome.

## Evaluation of the isPO-training (service providers’ perspective)

### Introduction to isPO and role-specific training

Two types of training were offered to the service providers: (1) introduction to isPO and care levels 0 and 1, and (2) training courses on care levels 2 and 3.

The two training sessions on the “introduction to isPO” and the care levels 0 and 1 were evaluated by 21 participants (response rate: 87.5%). The evaluations of the items on the comprehensibility of the contents were predominantly positive (mean values between 2.89 and 3.59 of 4) (see Additional file 4). In particular, the basic structure of the project was conveyed in an understandable way. However, the case managers' area of responsibility with regard to onco-guide care was the least comprehensible. The items concerning the trainers and the training organisation were also rated highly positively, with the time taken and the clarification of open questions rated on average at 2.57 and 3.00 points, respectively.

The high complexity of the project was emphasised in the free text field of the questionnaire, and a resulting confusion and incomprehensibility of the training was described. It was noted that too much information was passed on to the participants in a too short time. Furthermore, the suggestion was made to focus the training more on practical information. Many questions had remained open.

The training courses on care levels 2 and 3 (*n* = 7; response rate: 53.8%) were predominantly rated "totally agreed" in the organisational aspects (see Additional file 5). The mean values for the training contents also range between 3.17 and 3.67 points. In particular, the contents regarding level 3 (psychotherapeutic psycho-oncological care) were assessed as being comprehensibly conveyed.

### isPO onco-guide training

Nineteen participants (former cancer patients) of two different onco-guide training sessions contributed to the evaluation (response rate: 100%). All items on the training content had average values ≥ 3.5 (highest value on the scale: 4) (see Additional file 6). Participants of the first of the two sessions agreed strongly that the training on theoretical contents (onco-guide concept, isPO programme) was sufficient.

In the open question, it was suggested that role playing that was performed to train the conducting of conversation should be carried out with an observer or in front of the whole group. At the second training session conducted two weeks later, the participants gave a particularly positive assessment of the content relevant to practical work as an onco-guide. It was suggested that the training should include content on the tasks of the case manager, social services and the psychosocial specialist (as a differentiation from the onco-guide) as well as general information on "types of cancer". The organisation of the training was similarly positively evaluated. The corresponding items achieved a mean value of > 3.3.

### CAPSYS^2020^ training

For the analysis of the CAPSYS^2020^ training evaluation the data of only one training session were available (*n* = 5, response rate: 83.3%), as the evaluation team was not informed about all planed and conducted course dates.

For all items, average values of  > 3.0 (with a highest possible value of 4) were found (see Additional file 7). Six of the nine items were rated by all participants as "totally agree". These relate to the training contents according to the application of CAPSYS^2020^ as well as to the assessment of the trainers and the training organisation. In comparison, the item "All my questions were clarified in the training" has the lowest value of 3.40. In the open text field, it was noted that the training could have taken place at a slightly earlier point in time before implementation. A more precise time window was not specified.

The results and findings of the prospective evaluation were fed back to the project leader and/or consortium partners, so that the isPO programme is continuously being optimised. Furthermore, a written report on the outcome of the prospective evaluation was submitted to the project leader, so all results were available for programme and implementation optimisation (Fig. 4).

## Discussion

**Fig. 9:**
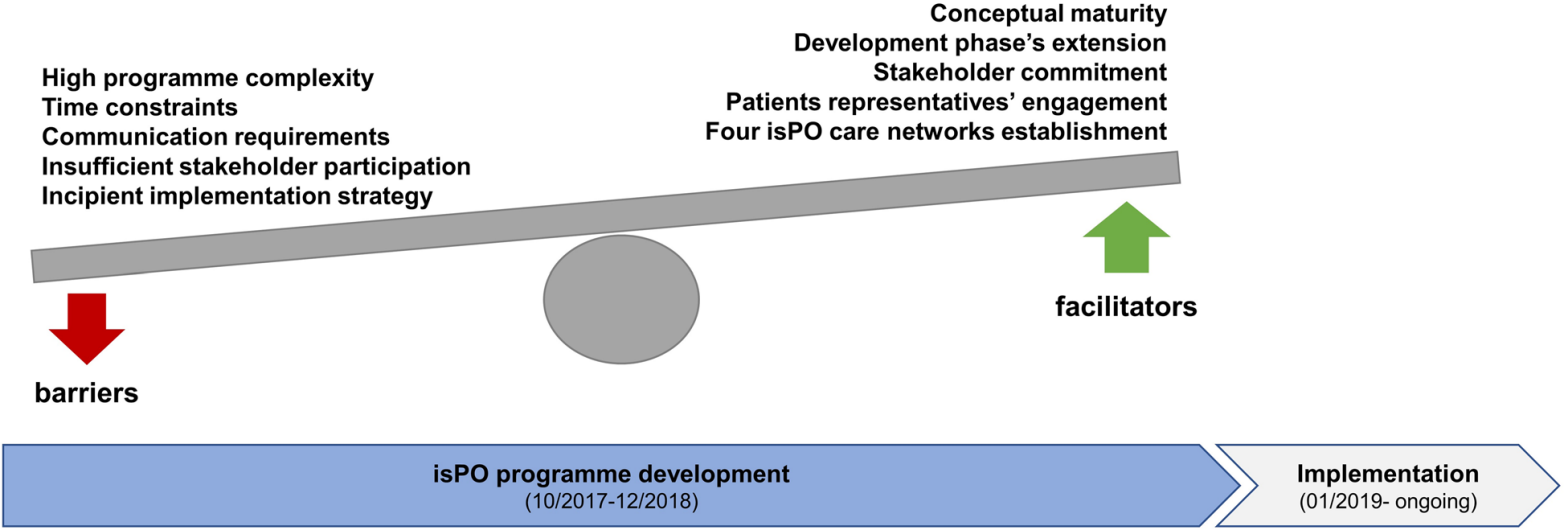
Condensed mixed-methods results of isPO’s prospective evaluation, representing end-users’, programme designers’ and service providers’ perspectives

During the isPO programme’s development phase, the evaluation team conducted a prospective evaluation with a QUAL-quant mixed-methods design. It assisted in gaining deep insight into the programme designers’ developing and working processes (for a condensed presentation of the results see Fig. 9) and the maturity of the programme.

In this respect, the prospective evaluation research questions (see introduction) are answered in the following and implications for other projects are given.

### Contextual challenges

#### Programme development

At the end of the first project year, the components of the isPO programme have reached a level of maturity that allows its implementation. However, its complete development could not be finished without three additional months (see results, chapter 'document analyses and interview with the project leader'). At this moment we were certain that the optimisation and finalisation of the components could take place in parallel to the implementation without impairment at the end-user or service provider level. Thus, the service providers’ experiences with the new programme can directly support its further development and optimisation processes. The particularly innovative components include, for example, the comprehensive quality management that enables a quality-based implementation and optimisation as well as the IT system, which serves not only for documentation but also for care management.

#### Recruitment and development of the isPO care networks

The four care networks were recruited and developed as anticipated in the project plan. The selection of the cancer centres was guided by criteria instead of randomised cluster sampling [29]. The participating hospitals are structurally diverse in, for example, bed capacity and number of organ centres within the cancer centres. Due to the different structures, processes, and organisational cultures, the developmental states vary between the care networks.

The development of the care networks has been carried out through time-consuming meetings. However, it benefited from the participation of different stakeholders from psycho-oncological and medical care, quality management and IT, as well as out-patient oncological care providers [30].

In some care networks concerns were repeatedly expressed about insufficient voluntary participation of physicians in this process. That might be due to the fact that psycho-oncological care has low priority in their daily routine, and to frequent medical staff turnovers. The isPO project management and the network developers were informed of this and meetings with the responsible hospital managers and medical directors were scheduled again.

#### Communication and cooperation of the programme designers

All consortium partners mutually appreciate the high level of commitment with which the isPO project is being developed. However, the interdependencies of the individual and emerging task areas and strong time constraints were perceived as challenging.

The lack of cooperation between designers and service providers was regretted. The programme’s contextual maturity would benefit from including expertise of real-world care practice, as it has also been experienced by other researchers [31–33].

The communication flow was found to be insufficient. However, in complex interventions, communication is central for the implementation’s success and therefore must be also integrated in the implementation strategies [34]. In our case, the programme designers’ suggestion to set up a superordinate communication body within the project management team is welcomed by the evaluation team, as an elaborated communication structure between project partners is crucial in a complex intervention project [35]. Even though tensions were not directly described, the limited time and human resources, the interdependencies and unclear lines of communication can be seen as risk factors. In addition, working with multidisciplinary partners requires mutual understanding [36]. In addition, all isPO consortium partners bring in their individual experience from previous projects and have partly already worked together, so that perhaps old conflicts are carried forward. This means that project management also needs to create mutual trust and offer opportunities for joint and co-creative learning and finding a collective language [37, 38]. The inclusion of the patient representatives’ perspective on the programme development (e.g. onco-guide conceptualisation) was beneficial, as therefore patients’ voice (e.g. opinion and expectations) was integrated. Patient involvement in the development process has also been suggested by other researchers, e.g. O'Cathain et al. [32].

#### Implementation facilitators and barriers, and implementation strategy

A positive attitude of the service providers and the clear concept of the isPO programme were considered as the two most important implementation facilitators, which is in line with the framework of actions for intervention development [32].

The programme designers regarded the lack of valuing the attitude and motivation of the stakeholders as obstacles in the implementation of isPO. It is therefore remarkable that the programme designers did not enter into an exchange with the service providers during the development phase (see RQ 3). In order to increase programme comprehensibility, and therefore acceptance, it would have been beneficial to involve all stakeholders in designing and refining the programme [32].

It was not anticipated that the design of the isPO programme itself could contain barriers. This may explain why few concrete suggestions were discussed for implementation outside of the project plan, such as the establishment of a helpdesk.

It has become apparent that during development of the programme components it was not considered in detail how they should be implemented, and thus no elaborate and context-specific implementation strategy was available. However, implementation strategies, including communication and providing feedback, are important for complex intervention programmes [34].

#### Consistency and usability of the isPO care programme

The complexity of the isPO programme was repeatedly challenging due to the limited amount of time, unexpected additional work required, and the coordination of the programme designers’ task areas and communication. The delayed completion of the programme’s development had a negative impact on the service providers in that the training sessions took place later than intended. As a further consequence, the service providers criticised that the training content was presented in a very compressed form. A lack of in-depth knowledge of processes and the resulting difficult working conditions could reduce service providers' acceptance towards the new programme and make the implementation more difficult. Better articulation of the programme idea [32] may reduce resistance.

Even if the interdependencies were experienced as a challenge, it demonstrates that the isPO components were developed in the sense of a coherent complex programme [16].

The patients’ representatives (HKSH-BV) also evaluated the isPO programme as sustainable, especially because of the stepped care approach [14], which pursues needs-based care and thus considers health economic requirements by preventing overprovision.

With regard to the characteristic ‘integrated’, isPO is particularly innovative, as it contains the integration of self-help and psychosocial care as fixed, contractually anchored, and financed care components, which currently are not part of the German Code of Social Law. In addition, the isPO programme, with its cross-sectoral approach, overcomes sector boundaries and hence facilitates the reduction of care interruptions and safeguards care quality [39].

Altogether, the prototype of a scientifically based care programme for the psycho-oncological care of newly diagnosed cancer patients was developed, which for the most part fulfils the requirements for successful implementation.

#### Implications for other projects

Conducting a prospective evaluation, i.e. identifying facilitators and barriers and assessing the suitability and the maturity of health programmes prior to their implementation, helps to avoid research waste [40] and harm through research and interventions [41]. This is possible by (1) increasing the maturity and comprehensibility of the programme, (2) ensuring its implementability in the health care system, and (3) gaining a sound understanding of each stakeholder’s needs [42, 43]. Thus, a prospective evaluation offers the benefit of supporting a smooth implementation [42] so that patient recruitment is easier and that the intervention is delivered by the service providers as intended [44]. In this way, subsequent follow-up costs for optimisation measures can be saved and the effectiveness and quality of the health programme can be enhanced [45]. Thus, a prospective evaluation reduces uncertainty about a programme's degree of success for all stakeholders: implementers, evaluators, but also funders and patients.

Following the reported findings, a prospective evaluation can be recommended especially for complex interventions [20].

### Methodological discussion

#### Conducting a process-oriented prospective evaluation

Prospectively evaluating a complex intervention programme was perceived as challenging. Because the respective care programme is a prototype that is not implemented, end-users or service providers cannot be asked about their experience with the new programme. Therefore, the prospective evaluation needs to be process-oriented, which is also emphasized in research regarding the development phase of complex interventions [46]. However, there are currently no exemplifying publications for comprehensive prospective evaluation concepts (before implementation).

Beside the fact that the funder required an external evaluation of the isPO-programme, investing in a comprehensive prospective evaluation was experienced as highly relevant by both the evaluation team itself and the programme designers. Before the implementation of the programme into practice possible facilitators and barriers were identified and a sound understanding of the programme’s development from an external perspective was gained. Findings were actively fed back to the project leadership which might positively influence the implementation. Moreover, the prospective evaluation may support the entire evaluation process (e.g. exploring the outcome) of a complex intervention, as its structured, systematic approach offers an in-depth programme understanding (e.g. components, stakeholders, context) [21, 47]. However, due to the role of the external evaluator, and how it was set in the project plan, dissemination concerning the evaluation results by the evaluation team were mostly provided to the project leader who then decided if it should be forwarded to the other consortium partners. This top-down approach hinders or impedes communication and constructive feedback transmission (selection bias). Direct and independent feedback to those who are involved in the process may be even more effective for the programme development, as suggested by Moore et al. [20].

#### Limitation and strengths of applying a mixed-methods evaluation design

The evaluation team’s findings show that applying a mixed-methods design seems to be crucial when aiming to evaluate a programme’s development, as recommended by other researchers [21, 48]. In particular, by collecting different kinds of qualitative data it was possible to obtain rich records on different aspects of the development phase [49]. Hereby, qualitative data (focus group and telephone interview and document analyses of the QPRs) were the most valuable data sources for conducting the prospective evaluation.

The focus group assisted in identifying “blind spots”, e.g. insufficient implementation strategies. Moreover, it allowed the evaluation team to interact with the programme developers, and therefore to gain a better impression of the cooperation and interpersonal aspects which are important factors in successfully developing and implementing a programme. Nevertheless, the focus group and telephone interview took place only once, so there is limited data on the course of the development. Therefore, a total of 15 QPRs were analysed.

This helped to systematically explore how the designers dealt with project and work plan deviations. However, the development of a QPR evaluation system turned out to be important (Table 1) in order to recognize critical aspects and to track the course of development. The criteria catalogue presented in this article may provide a good basis for other programme process-oriented evaluations, as it includes many criteria on work progress itself, and allows isPO-programme-specific criteria to be altered for respective usage. Additionally, the regular receipt of the QPRs allows evaluators to be more “in the loop” and give systematic and timely feedback to the project leaders. However, the QPRs did not fully reflect the cooperation and communication between the project partners, which could be captured by the beforementioned interviews.

#### Gathering the patients’ perspective in the prospective evaluation

The patient perspective was included in our prospective evaluation as proposed by different researchers [32, 47] by including participants of the HKSH-BV in the focus group. This was crucial for the prospective evaluation, as little attention was given to the patients’ perspective during the isPO programme’s development. Development was carried out top-down with a low degree of patient participation, as the HKSH-BV was limited to its advisory role.

### Lessons learned

#### Resources

The estimate of a one-year timescale for the development of such a complex care programme as isPO proved to be too tight. Enabling and managing the communication between the numerous project stakeholders appeared to be challenging, thus, the development of a project communication strategy would have been vital [34, 35]. Moreover, the establishment of the legal and ethical framework within the development phase was challenging in terms of time and effort. This is because the structures of the isPO programme go beyond the current legal situation, and the EU General Data Protection Regulation came into force. It is important to consider a realistic timeframe for setting up a contractual and ethical framework, because the start of a programme’s implementation is highly dependent on this.

#### Implementation strategy

A mature implementation strategy was not developed but would be highly beneficial [34]. Moreover, to develop a programme suitable for everyday care with regard to its feasibility and to tailor its implementation by considering the characteristics of the care organisations, two measures would have been helpful: (1) stakeholder analysis and (2) use of a participatory approach at selected points in the development phase [31, 50]. Simply transferring knowledge, as is the case with the conducted training sessions, appeared to be insufficient and needs to be augmented by on-the-job training in the care organisations [32]. Furthermore, if sufficient resources are available, the conduction of single or focus group interviews with (former) patients is recommendable to obtain information on key aspects that need to be considered in the development of a new intervention programme.

#### A process-oriented evaluation design

Complex interventions benefit from a process-oriented evaluation design that starts in the development phase. For a mature prospective evaluation concept with specifically defined outcomes we found it helpful to orientate towards national requirements and make use of mixed-methods to obtain rich data and gain deep insight into the programme’s development process. In addition to the QPRs, a focus group with representatives of all involved programme designers is especially recommendable in order to gain rich insight into attitudes, communication processes, and to identify blind spots, e.g. with regard to implementation strategies. Furthermore, the project leader has an extensive overarching understanding of the complex programme. Therefore, an interview with project leadership, which usually equals project management, allows deep insight into the programme concept beyond the project proposal.

#### Dissemination of evaluation results

In a complex intervention programme with a narrow timeframe it could be more manageable, helpful, and practice-oriented to give regular feedback to project leadership about important or urgent evaluation results. This helped project management in coordinating the programme’s development and gave programme designers the opportunity to find solutions for their blind spots. Still, the prospective evaluation report (at the end of the programme’s development phase) includes more detailed results and a thoroughly written up conclusion, lessons learned and recommendations which allow them to be put into context and enable further action, if necessary.

## Conclusions

By the end of the development phase, the fundaments for the programme’s implementation in practice for four German psycho-oncological isPO care networks had been laid.

As the development of a complex care programme is considered as crucial for a programme’s implementation success, the programme development should not be underestimated in terms of resources, e.g. finances, and availability of sufficient and skilled personnel. Furthermore, a realistic timeframe is essential, as the coordination of and communication between the various consortium partners needs to be addressed. A complex programme invests in developing comprehensive project management and context-specific implementation strategies as well as participatory involvement of the service providers and patients to facilitate practice-oriented programme optimisations.

A systematic mixed-methods approach turned out to be fruitful in evaluating a complex care programme prospectively. During the development phase, besides producing a final prospective evaluation report, proactively providing regular and critical feedback from the evaluation team is supporting the development process with regard to its implementation in practice.

The prospective evaluation should be given more importance in the research of healthcare programmes to prepare tailored implementations. In addition to the evaluation design, the prospective evaluation should also be considered in the publication plan, as this allows all evaluation phases to be retraced.

## Supplementary Information

**Additional file 1:** List of consortium partners conducting the isPO project

**Additional file 2:** Coding system for the analyses of the focus group and telephone interview

**Additional file 3:** Aims of the development phase concerning each programme component and their actual achievements

**Additional file 4:** Descriptive statistics for each item of the basic training evaluation ‘introduction to isPO and care levels 0 and 1’

**Additional file 5:** Descriptive statistics for each item of the basic training evaluation ‘care levels 2 and 3’

**Additional file 6:** Descriptive statistics for each item of the isPO onco-guide training evaluation

**Additional file 7:** Descriptive statistics for each item of the CAPSYS^2020^ training evaluation

## Data Availability

The datasets generated and/or analysed during the current study are not publicly available due to ethical and legal restrictions but are available from the corresponding author on reasonable request.

## Abbreviations

C: Component of the isPO care programme
CAPSYS^2020^: IT documentation and assistance system for the isPO programme
CFIR: Consolidated Framework for Implementation Research
HKSH-BV: House of Cancer Patient Support Associations of Germany
IF: Innovation Fund of the Federal Joint Committee, Germany
IMVR: Institute of Medical Sociology, Health Services Research, and Rehabilitation Science
isPO: integrated, cross-sectoral psycho-oncology
MRC: Medical Research Council
QPR: Quarterly progress report
RQ: Research question
