# Supplementary material for: Conducting a prospective evaluation of the development of a complex psycho-oncological care programme (isPO) in Germany
Source: BMC Health Serv Res. 2022 Apr 22;22:531. doi: 10.1186/s12913-022-07951-1 (PMC9026657; doi:10.1186/s12913-022-07951-1)
Supplement: Supplementary file 1 — Additional file 1: List of consortium partners conducting the isPO project [file 12913_2022_7951_MOESM1_ESM.pdf]

Additional file 1

Table A.1. List of consortium partners conducting the isPO project.

Abbreviation	isPO consortium partner
MED I	Department of Internal Medicine, Section: Clinical Psycho-Oncology, Working Group Psycho-Oncological Health Services Research, University Hospital of Cologne
KPP	Clinical Psychology and Psychotherapy, University of Cologne
IMSB	Institute of Medical Statistics and Computational Biology, University of Cologne
IGKE	Institute of Health Economics and Clinical Epidemiology, University of Cologne
MIFH	Department of Computer Science (Medical Informatics), University of Applied Sciences and Arts Dortmund
IMVR	Institute of Medical Sociology, Health Services Research and Rehabilitation Science, University of Cologne
KGNRW	Cancer Society North Rhine-Westphalia
HKSH	House of Cancer Patient Support Associations of Germany
TK	Techniker Krankenkasse (statutory health insurance fund)
BARMER	BARMER (statutory health insurance fund)
AOK	AOK Rheinland/Hamburg (statutory health insurance fund)
