# Supplementary material for: Conducting a prospective evaluation of the development of a complex psycho-oncological care programme (isPO) in Germany
Source: BMC Health Serv Res. 2022 Apr 22;22:531. doi: 10.1186/s12913-022-07951-1 (PMC9026657; doi:10.1186/s12913-022-07951-1)
Supplement: Supplementary file 2 — Additional file 2: Coding system for the analyses of the focus group and telephone interview [file 12913_2022_7951_MOESM2_ESM.pdf]

Additional file 2

Table A.2. Coding system for the analyses of the focus group and telephone interview.

Core categories	Subcategories
Expectations of the isPO programme	
Cooperation of project partners	Communication
	Distribution of tasks
	Overarching cooperation of the project partners
	Workload
	Intensity
	Engagement of the project partners
	Dependencies
Cooperation with care networks	Communication
	Intensity
	Contents of the cooperation
Facilitators of implementation into care networks	Acceptance and motivation of the service providers
	Structure and quality assurance of the isPO programme
	Monetary incentive
	Patient orientation
	Care networks’ structure
	Preservation of therapeutic freedom
Barriers of implementation into care networks	Care networks’ negative attitude
	Increased expenditure
	Determination of included professions
	Structure of the care networks
	Study part of the isPO project
	Financial interests of the care networks
	Low patient orientation
	Ambiguities and uncertainties in the care networks
Implementation strategies	Dealing with resistance / non-execution of the isPO guidelines
	Contact person
	Communication paths
	Trainings
	CAPSYS
	Preparation of the concept contents
	Handouts

Core categories	Subcategories
Facilitators for implementation into conventional care	Patient interest
	Useful evaluation
	Political commitment
	Uniform care structure
Barriers for implementation into conventional care	Professional policy factors
	Bureaucratic hurdles
	Methodological weaknesses
	Unclear implementation framework
