# Supplementary material for: Conducting a prospective evaluation of the development of a complex psycho-oncological care programme (isPO) in Germany
Source: BMC Health Serv Res. 2022 Apr 22;22:531. doi: 10.1186/s12913-022-07951-1 (PMC9026657; doi:10.1186/s12913-022-07951-1)
Supplement: Supplementary file 3 — Additional file 3: Aims of the development phase concerning each programme component and their actual achievements [file 12913_2022_7951_MOESM3_ESM.pdf]

Additional file 3

Table A.3. Aims of the development phase concerning each programme component and their actual achievements. If aims have been met in full, they are indicated with a check mark (✓) in the achievement column.

Components	Aims within development phase	Actual achievements at the end of development phase
**C1: Care concept**	• **development of a care concept** based on/compliant with<br>- programme theory [1]<br>- “best practice” model [2]<br>- S3 guideline for psycho-oncology [3]<br>→ requirements:<br>- developing effect theory for each care level<br>- evidence summary on genesis of psychological and psychosocial distress in cancer patients, associated consequences and suitable interventions<br>- description on how care should be provided according to patients' needs at different care levels<br>• **providing a treatment manual** as translation of isPO care concept into practical psycho-oncological care	• **development of scientific basis of the care concept**<br>- effect theory developed<br>- screening instruments for assigning patients to care levels developed<br>• **first version of treatment manual available**
**C2: Project & care management**	• **development of document control system**: contains clear descriptions for all contract-related processes (C4), defining activities, responsible persons, times, etc. (C1, C5, C6)	• **creation of database and document control system** based on an Excel file that was made available to all care networks (C3)<br>• **further activities:**<br>- provision of a milestone plan with overview on the provision and delivery of results and products in all task areas to all consortium partners

Components	Aims within development phase	Actual achievements at the end of development phase
		- continuous and regular meetings between project leader and consortium partners<br>- establishment of semi-annual steering committee meetings with all consortium partners and representatives of care networks (C3)
**C3: Foundation, development, & support of isPO care networks**	• **foundation and development of four care networks**<br>→ requirements:<br>- recruiting three non-university care networks in addition to the University Hospital Cologne on the basis of criteria of (economic-)geographic, population structural and cross-sectoral psycho-oncological care structural characteristics<br>- training of service providers on the structure and functions of the care networks, on the stepped care concept (C1), on quality management (C5) and on the different care level processes depending on their role in the programme<br>- recruiting, training and certification of isPO onco-guides	• **four care networks have been founded and basic care structures have been developed**<br>✓<br>- training documents were produced and basic training with service providers was conducted → in-depth courses still to come<br>- isPO onco-guide teams for every care network have been recruited, trained and certified, but acquisition process is ongoing<br>• **further activities:**<br>- gathering a written overview of the current psycho-oncological care structures in each care network for designing the implementation process<br>- planning the set-up of a helpdesk to ensure that acute problems in isPO-related care provision are promptly and efficiently dealt with
**C4: Contracts & agreements**	• **signed consortium partner agreement**: regulating areas of responsibility of the consortium partners and their cooperation (C2)	✓

Components	Aims within development phase	Actual achievements at the end of development phase
	• **four signed cooperation agreements**: specifying the tasks of the participating care networks in the context of care and the study (C3)<br>• **signed care contracts between the care networks and the participating health insurance companies**: regulating the obligations, requirements and services of the contractual partners, and the financing of psycho-oncological services (C3)	✓ **cooperation agreements have been signed by all parties involved**<br>✓<br>→ care contract meets the requirements of German social law
**C5: Quality management**	• **development of quality management concept** as it is required by German social laws and according to DIN EN ISO standards for quality management especially for health care organisations<br>• **conceptual design of intra- and inter-institutional quality assurance measures** (C3 & C4) (participatory quality development approach)	• **a beta version of the isPO QM manual has been produced**<br>✓
**C6: Care pathways & indicators**	• **development of SOPs** operationalising care concept (C1) and care management (C2):<br>- specification who, why, which service should be performed in patient care ("doing the right thing")<br>- definition of how and when, and the goal of the activity ("doing the right thing right")<br>→ SOPs also basis of the programming of CAPSYS^{2020} (C7)<br>• **modulation of SOPs as treatment paths** for integration into quality management (C5) during the course of the project<br>• **development of quality indicators** (see also C8)	• **development of basic SOPs for care levels 0 to 3a**<br>• **modulation of care pathways for care levels 0 to 2**

Components	Aims within development phase	Actual achievements at the end of development phase
		• **quality indicators have not been developed so far**, as the methodological approach planned appeared to be inappropriate and was adapted
**C7: IT-documentation & assistance system**	• **development of the computer-based documentation and assistance system CAPSYS^{2020}** with the following functions:<br>- structured care management (C2, C6)<br>- automatic reporting for QM (C5)<br>- accounting functions for controlling (C4)<br>- patient-related and cross-patient planning, documentation, monitoring, and evaluation of care provision (C1, C8)<br>- supporting functions according to different service provider roles (C1, C3)<br>- processing of cancer registry data (C8)	• **development of CAPSYS^{2020} completed for the following functions**:<br>✓<br>-<br>-<br>✓<br>✓<br>-
**C8: Evaluation**	• **programming of care statistics** based on quality indicators (C6) for automatic quality reports (C5) as a function of CAPSYS^{2020} (C7)<br>• **setting up a data warehouse** for controlled and secure transfer of isPO care data (C7) to evaluating institutes (KPP, IGKE, IMVR)<br>• **developing a data protection concept**	• **derivation of test quality indicators** from the cooperation agreements (due to delay in C6)<br>✓<br>✓<br>• **further activities:**<br>- the health insurance companies involved in the project requested a health economic evaluation after project beginning that had to be implemented additionally

References

1. Issel LM, Wells R. Health program planning and evaluation: A practical, systematic approach for community health. Burlington, MA: Jones & Bartlett Learning; 2017.
2. Kusch M, Labouvie H. Anwendungsorientierte Versorgungsforschung in der Psychoonkologie. 2015. https://www.researchgate.net/publication/312291822_Anwendungsorientierte_Versorgungsforschung_in_der_Psychoonkologie. Accessed 3 Sept 2021.
3. Leitlinienprogramm Onkologie. S3-Leitlinie Psychoonkologische Diagnostik, Beratung und Behandlung von erwachsenen Krebspatienten. 2014. https://www.leitlinienprogramm-onkologie.de/fileadmin/user_upload/Downloads/Leitlinien/Psychoonkologieleitlinie_1.1/LL_PSO_Langversion_1.1.pdf. Accessed 3 Sept 2021.
