# Supplementary material for: Conducting a prospective evaluation of the development of a complex psycho-oncological care programme (isPO) in Germany
Source: BMC Health Serv Res. 2022 Apr 22;22:531. doi: 10.1186/s12913-022-07951-1 (PMC9026657; doi:10.1186/s12913-022-07951-1)
Supplement: Supplementary file 4 — Additional file 4: Descriptive statistics for each item of the basic training evaluation ‘introduction to isPO and care levels 0 and 1’ [file 12913_2022_7951_MOESM4_ESM.pdf]

Additional file 4

Table A.4. Descriptive statistics for each item of the basic training evaluation ‘introduction to isPO and care levels 0 and 1’.

Item	Valid cases	Missings	M	SD	Min	Max
The training provided a comprehensible introduction to...						
... the fundamental structure of the project.	21	0	3.57	0.51	3	4
...the concept of the isPO care form.	21	0	3.24	0.77	2	4
...the structure and function of the care networks.	21	0	3.05	0.74	2	4
...the care procedures in the care levels.	21	0	3.10	0.70	2	4
...the quality management system.	20	1	3.05	0.60	2	4
...the procedures for patient information and enrolment.	20	1	3.10	0.72	2	4
... the onco-guide concept.	20	1	3.35	0.59	2	4
...my task area in the onco-guide's care.	17	4	2.88	0.86	1	4
All my questions were answered during the training.	18	3	2.78	0.73	2	4
The time frame of the training was appropriate.	20	1	2.70	0.80	1	4
The trainers were competent.	21	0	3.76	0.44	3	4
The trainers were motivated.	19	2	3.74	0.45	3	4
The training was well organised.	21	0	3.43	0.60	2	4
Overall, I am satisfied with the training.	21	0	3.24	0.70	2	4
