# Supplementary material for: Conducting a prospective evaluation of the development of a complex psycho-oncological care programme (isPO) in Germany
Source: BMC Health Serv Res. 2022 Apr 22;22:531. doi: 10.1186/s12913-022-07951-1 (PMC9026657; doi:10.1186/s12913-022-07951-1)
Supplement: Supplementary file 5 — Additional file 5: Descriptive statistics for each item of the basic training evaluation ‘care levels 2 and 3’ [file 12913_2022_7951_MOESM5_ESM.pdf]

Additional file 5

Table A.5. Descriptive statistics for each item of the basic training evaluation ‘care levels 2 and 3’.

Item	Valid cases	Missings	M	SD	Min	Max
The training provided a comprehensible introduction to...						
...the concept of the psychosocial care.	7	0	3.43	0.53	3	4
...the procedures of the psychosocial care.	7	0	3.29	0.49	3	4
...the evaluation of psychosocial support needs.	7	0	3.43	0.53	3	4
...the concept of the psycho-oncological-psychotherapeutic care.	7	0	3.43	0.79	2	4
...the procedures of the psycho-oncological-psychotherapeutic care.	6	1	3.67	0.52	3	4
...the structure of the intervention modules of the isPO manual.	6	1	3.17	0.41	3	4
...the concept of the complex psycho-oncological care (level 3b).	6	1	3.33	0.82	2	4
...the procedures of the complex psycho-oncological care (level 3b).	6	1	3.50	0.55	3	4
All my questions were answered during the training.	6	1	3.50	0.55	3	4
The time frame of the training was appropriate.	7	0	3.71	0.49	3	4
The trainers were competent.	7	0	3.86	0.38	3	4
The trainers were motivated.	7	0	4.00	0.00	4	4
The training was well organised.	7	0	3.86	0.38	3	4
Overall, I am satisfied with the training.	7	0	3.86	0.38	3	4
