# Supplementary material for: Conducting a prospective evaluation of the development of a complex psycho-oncological care programme (isPO) in Germany
Source: BMC Health Serv Res. 2022 Apr 22;22:531. doi: 10.1186/s12913-022-07951-1 (PMC9026657; doi:10.1186/s12913-022-07951-1)
Supplement: Supplementary file 6 — Additional file 6: Descriptive statistics for each item of the isPO onco-guide training evaluation [file 12913_2022_7951_MOESM6_ESM.pdf]

Additional file 6

Table A.6. Descriptive statistics for each item of the isPO onco-guide training evaluation.

	Session 1						Session 2					
**Item**	**Valid cases**	**Missings**	**M**	**SD**	**Min**	**Max**	**Valid cases**	**Missings**	**M**	**SD**	**Min**	**Max**
There has been sufficient training on...												
...the isPO care programme.	8	1	4.00	0.00	4	4	10	0	3.60	0.52	3	4
...the onco-guide concept.	9	0	3.78	0.44	3	4	10	0	3.60	0.52	3	4
...the contents and use of the onco-guide information package.	9	0	3.56	0.53	3	4	10	0	3.60	0.52	3	4
...the task area of the onco-guide	9	0	3.78	0.44	3	4	10	0	3.70	0.48	3	4
...the conversational technique of an onco-guide.	8	1	3.38	0.52	3	4	10	0	3.80	0.42	3	4
...the documentation of the onco-guide meeting.	9	0	3.56	0.53	3	4	9	1	3.89	0.33	3	4
... data protection and confidentiality.	9	0	3.78	0.44	3	4	10	0	3.90	0.32	3	4
...the transition from certification to deployment in the care networks.	8	1	3.63	0.52	3	4	10	0	3.40	0.70	2	4
All my questions were answered during the training.	9	0	3.56	0.73	2	4	9	1	3.33	0.50	3	4
The time frame of the training was appropriate.	9	0	3.56	0.53	3	4	10	0	3.70	0.48	3	4
The trainers were competent.	9	0	3.78	0.44	3	4	10	0	3.90	0.32	3	4
The trainers were motivated.	9	0	3.78	0.44	3	4	10	0	3.90	0.32	3	4
The training was well organised.	9	0	3.89	0.33	3	4	10	0	3.90	0.32	3	4
Overall, I am satisfied with the training.	9	0	3.78	0.44	3	4	10	0	3.80	0.42	3	4
