# Supplementary material for: Conducting a prospective evaluation of the development of a complex psycho-oncological care programme (isPO) in Germany
Source: BMC Health Serv Res. 2022 Apr 22;22:531. doi: 10.1186/s12913-022-07951-1 (PMC9026657; doi:10.1186/s12913-022-07951-1)
Supplement: Supplementary file 7 — Additional file 7: Descriptive statistics for each item of the CAPSYS2020 training evaluation [file 12913_2022_7951_MOESM7_ESM.pdf]

Additional file 7

Table A.7. Descriptive statistics for each item of the CAPSYS^{2020} training evaluation.

Item	Valid cases	Missings	M	SD	Min	Max
The general usage of CAPSYS was explained comprehensibly.	5	0	4.00	0.00	4	4
The use of the functions that are relevant for me was explained comprehensibly.	5	0	4.00	0.00	4	4
The care management by CAPSYS was explained comprehensibly.	5	0	4.00	0.00	4	4
All my questions were answered during the training.	5	0	3.40	0.55	3	4
The time frame of the training was appropriate.	5	0	3.60	0.55	3	4
The trainers were competent.	5	0	4.00	0.00	4	4
The trainers were motivated.	5	0	4.00	0.00	4	4
The training was well organised.	5	0	4.00	0.00	4	4
Overall, I am satisfied with the training.	5	0	3.80	0.45	3	4
